# Outcomes in Patients Undergoing Transoral Incisionless Fundoplication With Ineffective Esophageal Motility

**DOI:** 10.1155/grp/5054381

**Published:** 2025-12-22

**Authors:** Dhruvanshu Patel, Zarian Prenatt, Emilie S. Kim, Parampreet Kaur, Amit Sohagia, Vhada S. Sharma, Chandani Patel, Madhav Changela, Krutarth Shukla, Ronak Modi

**Affiliations:** ^1^ Department of Gastroenterology, St. Luke′s University Health Network, Bethlehem, Pennsylvania, USA, slhn.org; ^2^ Department of Research and Innovation, St. Luke′s University Health Network, Bethlehem, Pennsylvania, USA, slhn.org; ^3^ Gastroenterology, Geisinger Community Medical Center, Scranton, Pennsylvania, USA; ^4^ Pediatrics, St. Luke′s University Health Network, Bethlehem, Pennsylvania, USA, slhn.org; ^5^ Gujarat Adani Institute of Medical Sciences, Bhuj, Gujarat, India; ^6^ Internal Medicine, One Brooklyn Health System/ Interfaith Medical Center, Brooklyn, New York, USA, interfaithmedical.com; ^7^ Gujarat Cancer Society Medical College, Ahmedabad, Gujarat, India; ^8^ St Luke′s University Health Network, Bethlehem, Pennsylvania, USA, slhn.org

**Keywords:** gastroesophageal reflux disease, ineffective esophageal motility, proton pump inhibitors, transoral incisionless fundoplication

## Abstract

**Background:**

Transoral incisionless fundoplication (TIF) presents a minimally invasive, endoscopic treatment for the management of gastroesophageal reflux disease (GERD), known for its efficacy and safety in patients with normal esophageal motility. However, the use of TIF in ineffective esophageal motility (IEM) remains largely unexplored. This study examined the outcomes of TIF in IEM patients.

**Methods:**

Retrospective data from 164 patients, including 29 with IEM and refractory GERD, were analyzed to obtain assessments of disease‐specific quality of life through the Gastroesophageal Reflux Disease Health‐Related Quality of Life Questionnaire (GERD‐HRQL) and proton pump inhibitor usage.

**Results:**

Statistically significant improvements were seen in total, heart burn, and regurgitation GERD‐HRQL scores (*p* < 0.001, *p* < 0.001, *p* < 0.001) and reduction in PPI use (0 < 0.001) after TIF, with no significant difference in dysphagia risk (*p* < 0.2).

**Conclusion:**

This study underscores durable improvements that TIF can provide in quality of life in patients with both GERD and IEM, without compromising the increased risk of dysphagia. Although some patients resume antisecretory medications within 3–6 months, most stopped taking PPI after the procedure.

## 1. Introduction

Gastroesophageal reflux disease (GERD) is associated with reflux symptoms including heartburn, regurgitation, dysphagia, and various other extra‐esophageal symptoms [1–4]. It is also associated with an increased risk of cancer and reduced quality of life worldwide [1–4]. Although antireflux surgery has traditionally been the mainstay of treatment for GERD refractory to medical therapy, it is associated with various adverse effects, including dysphagia, flatulence, gas‐bloating syndrome, and surgical complications [1–4]. Transoral incisionless fundoplication (TIF) has emerged as a minimally invasive alternative for refractory GERD, demonstrating promising efficacy and safety profiles [1–4]. TIF works by reconfiguring the tissue to obtain a full‐thickness gastroesophageal valve from inside the stomach by serosa‐to‐serosa plications, which include the muscle layers. TIF is done with a partial anterior fundoplication with a wrap between 270° and 300°. The new valve is thought to restore the gastroesophageal flap, which would help barrier function from GERD. This procedure has potentially fewer side effects compared with surgery. TIF alone is adequate for a hiatal hernia (HH) size less than 2 cm (traditional TIF) and there is increasing data showing efficacy in performing laparoscopic HH repair surgery for patients with refractory GERD and HH size more than 2 cm prior to undergoing TIF, which is termed as concomitant transoral incisionless fundoplication (cTIF). Most studies have focused on outcomes in patients with normal esophageal motility, leaving a knowledge gap regarding the outcomes of TIF in individuals with minor esophageal motility disorders. Current guidelines recommend excluding patients with major esophageal motility disorders (like achalasia and hypercontractile esophageal motility disorders) from antireflux procedure candidacy, with only expert centers performing antireflux surgery in highly selected patients with absent primary peristalsis. However, TIF is only considered a relative contraindication in minor esophageal motility disorders such as ineffective esophageal motility (IEM). Our study aims to investigate the outcomes of TIF in patients with IEM. To the best of our knowledge, this study is the first of its kind to date.

## 2. Materials and Methods

### 2.1. Study Design and Participant Selection

**Figure 1:**
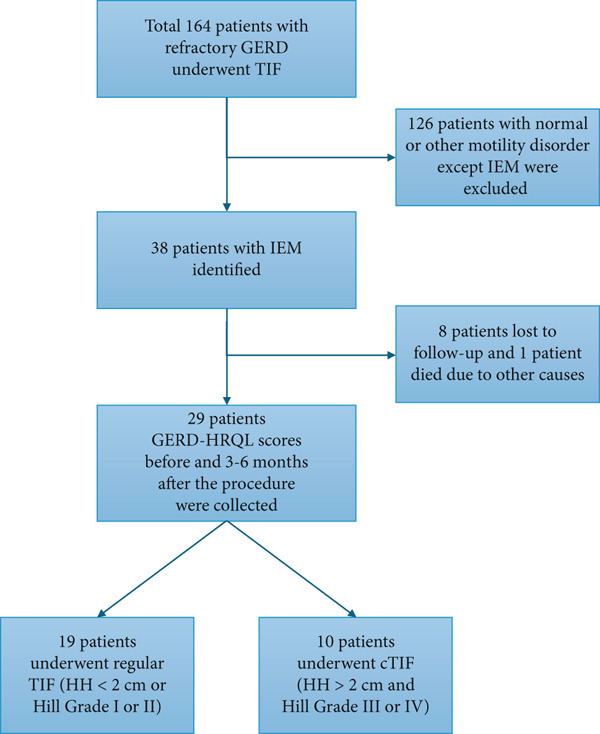
Study sample distribution.

We identified 164 participants with a long‐standing history of GERD refractory to acid suppressants who underwent TIF between January 2017 and December 2022 (Figure 1). They all had appropriate preoperative assessment including esophageal manometry, 24‐h pH study, and esophagogastroduodenoscopy (EGD). Of those patients, 29 were found to have IEM based on the Chicago Classification 3.0. IEM was defined as a distal contractile integral (DCI) less than 450 mmHg ^∗^s ^∗^cm on ≥ 50% liquid bolus swallows on manometry (Figure 1). We tried to determine the size of HH using EGD and manometry. Patients were classified based on Hill Classification where 19 patients with Hill Grade I or II HH size < 2 cm underwent traditional TIF and 10 patients with Hill Grade III or IV with HH size of > 2 cm underwent cTIF (Figure S1).

### 2.2. Inclusion Criteria

1. Age > 18 years,
2. On daily proton pump inhibitor (PPI) > 6 months,
3. Moderate to severe GERD symptoms based on the Gastroesophageal Reflux Disease Health‐Related Life Questionnaire (GERD‐HRQL) 10,
4. Esophageal manometry showing IEM,
5. Erosive esophagitis, and^1^
6. Abnormal ambulatory pH study.^2^

### 2.3. Exclusion Criteria

1. Age<18 years,
2. Esophageal manometry showing normal or other types of major or minor esophageal dysmotility except IEM, and
3. Erosive esophagitis LA Grade D.

### 2.4. Data Collection

Patients were retrospectively approached, and the GERD‐HRQL score was obtained through a telephone questionnaire, which assessed various aspects of GERD symptoms and their impact on daily life. The median follow‐up period after the TIF procedure was approximately 6 months. Patient demographics such as age, gender, body mass index (BMI), PPI use, and esophageal manometry showing IEM were identified using the electronic medical records (EMRs).

### 2.5. Operative Technique

TIF was performed using the EsophyX Z+ device^3^ (Figure 2, Figure 3, and Figure S2). Prophylactic antibiotics were given prior to the procedure. Traditional TIF was performed by an endoscopist and cTIF, where laparoscopic or robotic HH repair was performed by a surgeon, and then cTIF was done by the endoscopist. This device was inserted over a flexible endoscope into the stomach with the patient in the left lateral position under general anesthesia. We used a small endotracheal tube (6.5 or 7 mm) for intubation, considered to be more convenient for the endoscopists. Preoperative upper endoscopy was performed to assess the distance between the incisor teeth and the gastroesophageal junction (GEJ). We then determined the transverse dimension of the diaphragmatic hiatus. With the endoscope retroflexed for visualization of the GEJ, the device′s tissue mold was retroflexed (Figure 2a). From the apex of the device, a helical retractor was advanced and engaged into the tissue slightly distal to the Z line on the posterior wall. With caudal retraction on the helical retractor, the tissue mold was used to retract the fundus to bring it into apposition with the distal esophageal wall in order to create an esophagogastric plication (Figure 2b). The tissue mold and helix were locked in place, and polypropylene H‐shaped fasteners were deployed above the Z line so that one leg of the fastener engages in the esophageal lumen and the other in the gastric lumen (Figure 2c). This procedure was repeated by positioning the helical retractor once again into the tissue slightly distal to the Z line, this time on the anterior wall. Consequently, full thickness plications were created in two more sites, in between the posterior and anterior sites, until 20–30 fasteners were deployed in total. This resulted in an esophagogastric plication that extends above the Z line 2–4 cm (about 1.57 in.) and spans circumferentially 270°–300° (Figure 2d,e). All TIF procedures were performed by experienced and skilled endoscopists who first completed specific training on guinea pigs and subsequently performed six TIF cases under supervision before the start of this study.

Figure 2(a) Retroflexion view of GE junction with fundus, (b) EsophyX Z++ device with helical retractor engaged with GE junction at 11 o′clock position, (c) tissue arm mold closing to wrap stomach with GE junction, (d) anterior partial fundoplication wrapped with reconstructed GJ junction valve, and (e) fasteners made from polypropylene nonresorbable equivalent to 3–0 suture at GJ junction.

**Figure:**
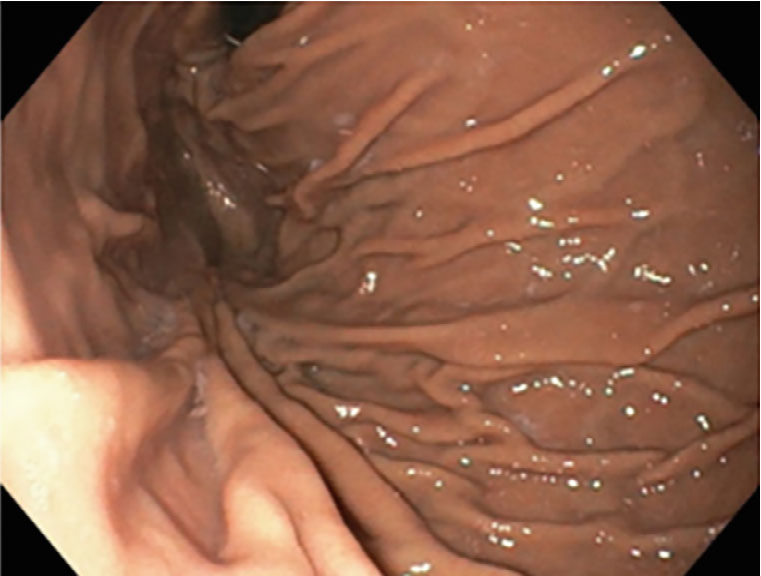
(a)

**Figure:**
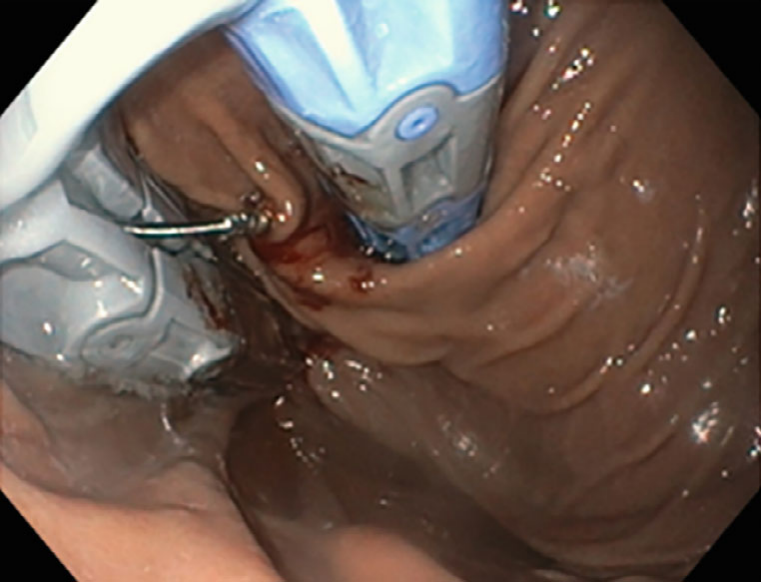
(b)

**Figure:**
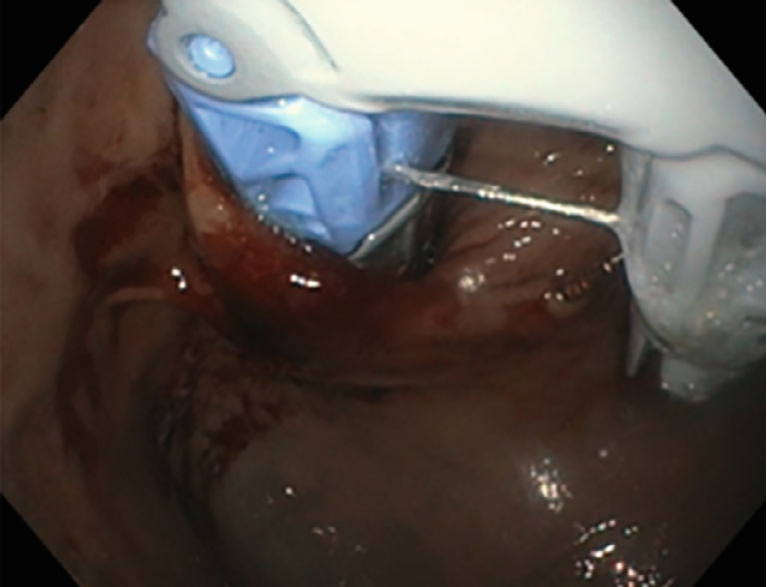
(c)

**Figure:**
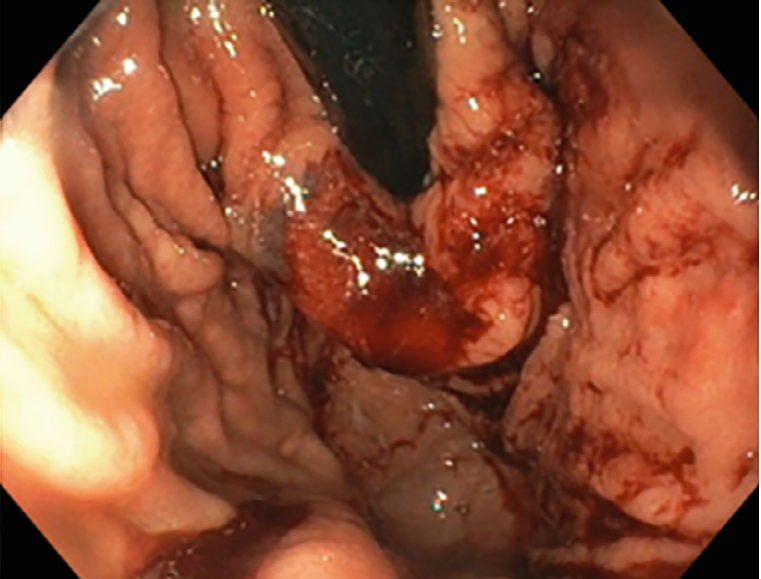
(d)

**Figure:**
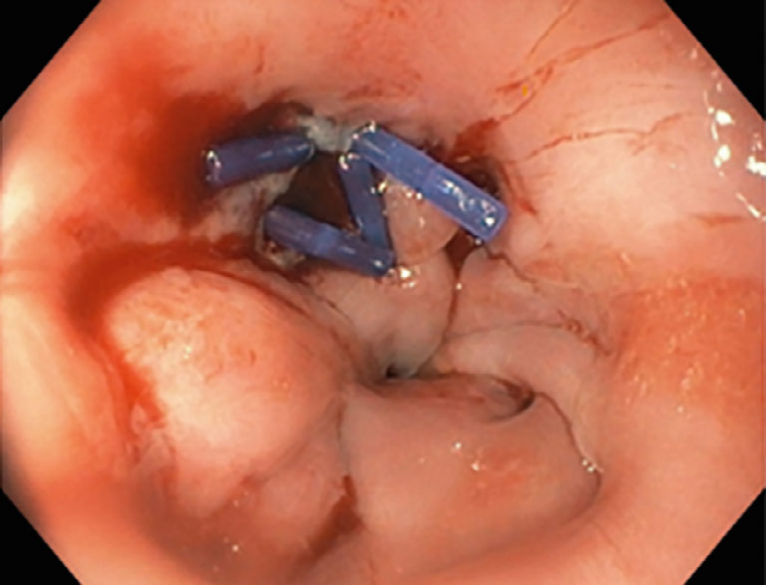
(e)

Figure 3Schematic representation of the procedure with EsophyX device (courtesy of EndoGastric Solutions Inc. Redmond, Washington, United States). (a) Endoscope retroflexed to visualize the fundus. (a) The device wraps the fundus around the distal esophagus and fastens a tissue fold. (c) and (d) This step is then repeated multiple times to reconstruct a robust, tight valve (2014 EndoGastric Solutions, Inc).

**Figure:**
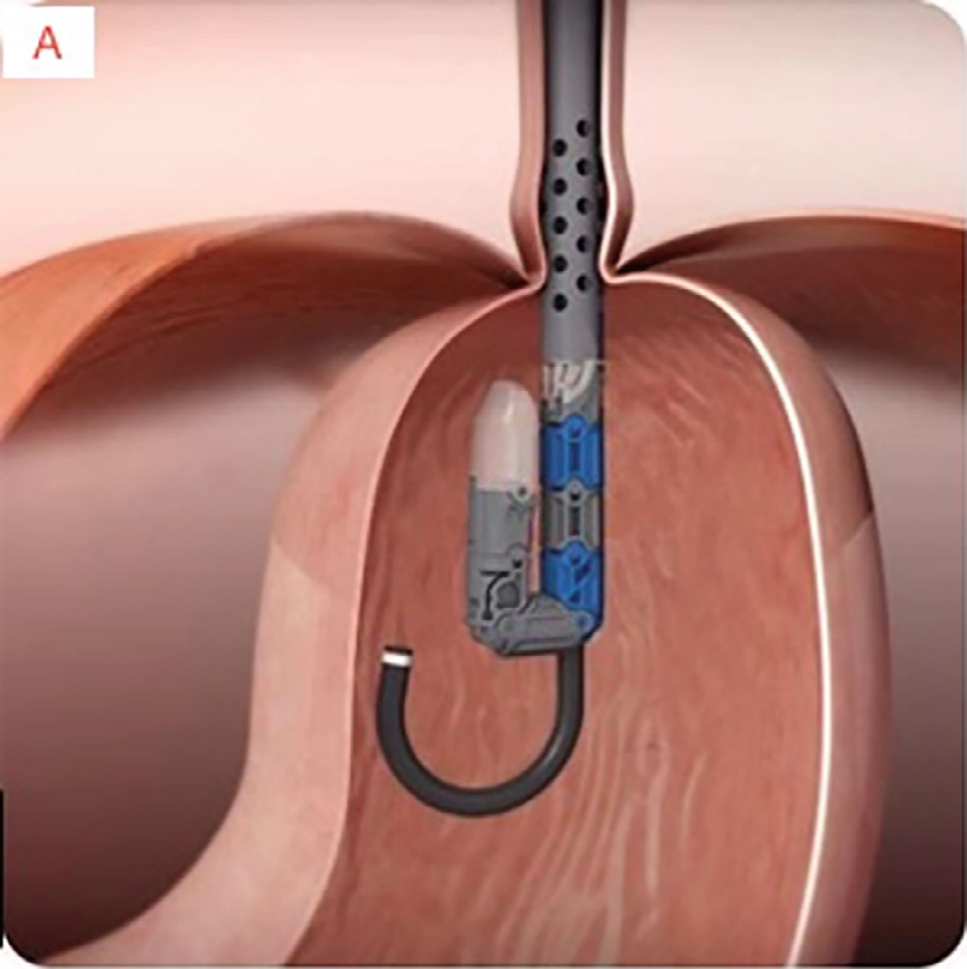
(a)

**Figure:**
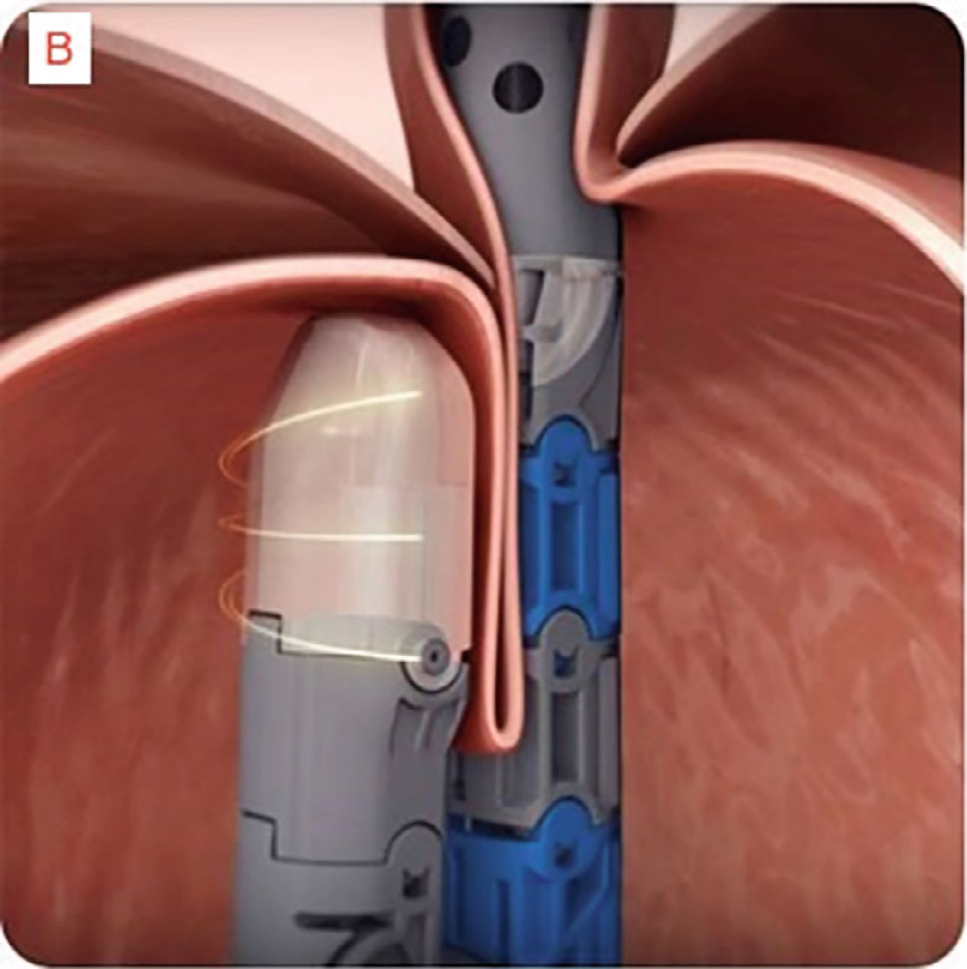
(b)

**Figure:**
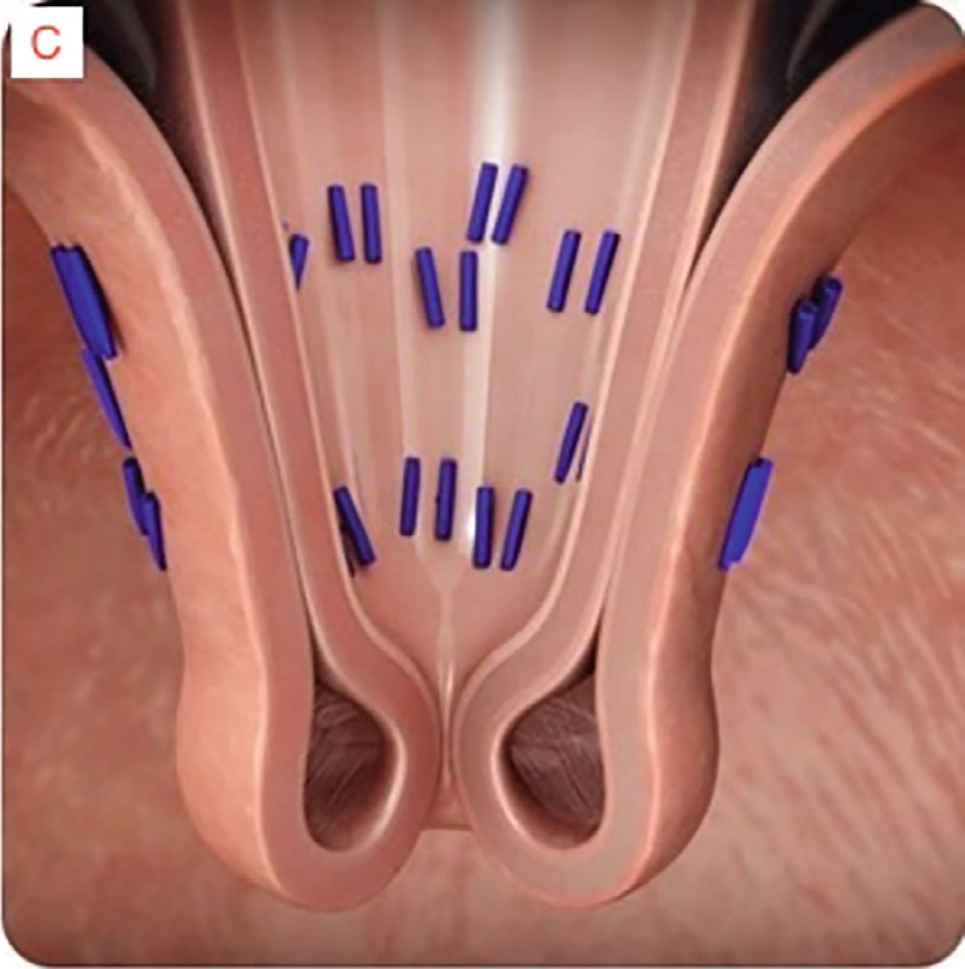
(c)

**Figure:**
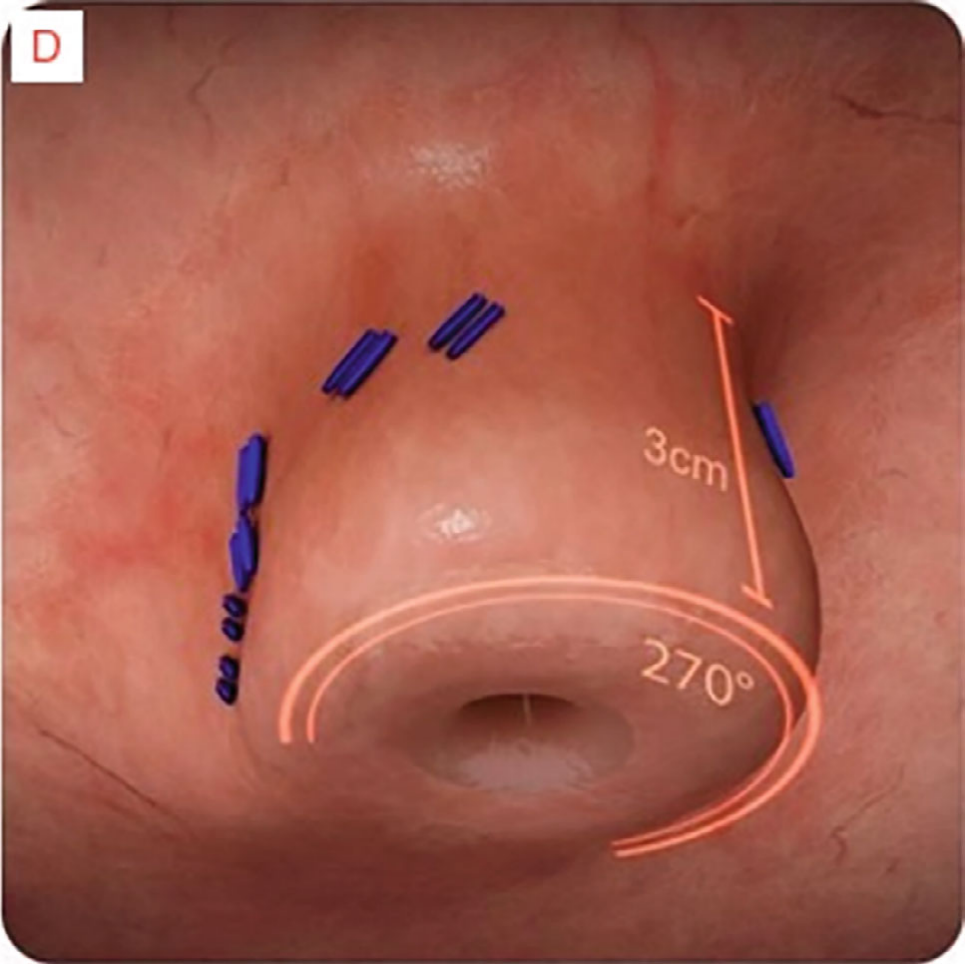
(d)

The incidence of serious adverse events was recorded. Serious adverse events were defined as complications needing hospitalization and medical or surgical intervention. Patients were discharged 2–4 days after the procedure and prescribed PPI therapy twice daily for the next 4 weeks to minimize the possibility of gastric bleeding and for healing of the traumatized esophageal tissue. Patients were thereafter asked to stop PPI therapy. They were instructed to follow a liquid diet for 2 weeks, a soft diet for another 4 weeks, and a normal diet thereafter. Postoperatively, patients were instructed to follow up at 1 month, 6 months, 12 months, and then annually for a 5‐year period.

### 2.6. Outcomes Measures

The primary outcome was measured by comparing GERD‐HRQL scores and evaluating the severity of heartburn, regurgitation, and dysphagia before and at 6 months following the procedure. The secondary outcome was measured by comparing the use and frequency of PPIs before and after TIF. We also conducted a subgroup analysis by dividing patients who had traditional TIF versus cTIF and comparing the improvement in GERD‐HRQL scores before and after the procedure.

### 2.7. Ethical Considerations

The study protocol was approved by the Institutional Review Board (IRB). Patient confidentiality and privacy were strictly maintained throughout the study, ensuring compliance with all relevant data protection regulations.

### 2.8. Statistical Methods

Statistical analyses were performed using the IBM SPSS Statistics 26 program. A Wilcoxon Signed Rank test was used for continuous variables, pre‐TIF and post‐TIF heartburn, regurgitation, dysphagia, and GERD‐HRQL scores for non‐normal distributions. Friedman tests were used for ordinal variables: pre‐TIF PPI use and post‐TIF PPI use. We also compared the subgroups based on the type of TIF procedure performed (traditional TIF vs. cTIF) by comparing the difference in improvement of GERD‐HRQL, heartburn, regurgitation, dysphagia scores prior to TIF and 6 months after TIF procedure by Mann–Whitney Tests.

## 3. Results

We retrospectively analyzed 29 patients with a median age of 68 (27–87) years. The female to male ratio was 1.9:1 with 65.5% females. The mean BMI was 29.2 ± std 5.1. TIF outcomes were assessed at 6 months postoperatively. There was a statistically significant improvement (*p* < 0.001) seen in total GERD‐HRQL, heartburn, and regurgitation scores, but dysphagia did not show any statistically significant difference (*p* < 0.2) (Table 1). There was a statistically significant difference between PPI use prior to TIF and at 6 months following TIF (*p* < 0.001) (Table 2). When comparing the patients who underwent traditional TIF (66%) to cTIF (33%), we found a statistically significant difference (*p* < 0.02) in improvement in GERD‐HRQL, heartburn, and regurgitation scores after the TIF procedure, with patients who had cTIF performed having even greater score reduction as compared with that of their counterparts. No significant difference in dysphagia (*p* = 0.8) was seen in both subgroups (Table 3). The incidence of serious and minor adverse events was recorded. Serious adverse events were defined as complications needing hospitalization and medical or surgical intervention.

**Table 1:** Comparison of pre‐TIF and post‐TIF GERD scores.

**GERD-HRQL score** **n** = 29	**Median score (minimum-maximum)**	**p** **(Wilcoxon rank test)**
Pre‐TIF total score (0–75)	35 (11–63)	< 0.001
Post‐TIF total score (0–75)	4 (0–25)	
Pre‐TIF heartburn score (0–30)	20 (0–29)	< 0.001
Post‐TIF heartburn score (0–30)	3 (0–11)	
Pre‐TIF regurgitation score (0–30)	17 (0–29)	< 0.001
Post‐TIF regurgitation score (0–30)	0 (0–12)	
Pre‐TIF dysphagia (0–5)	0 (0–5)	0.2
Post‐TIF dysphagia (0–5)	0 (0–2)	

**Table 2:** Comparison of pre‐TIF PPI use and post‐TIF PPI use (*p* < 0.001) (Friedman test).

**PPI use (number of days/week)**	**Pre-TIF PPI use frequency (percentage)**	**Post-TIF PPI use (3–6 months) frequency (percentage)**
None (0 days/week)	0 Patients (0%)	16 Patient (55.2%)
Occasional (1–3 days/week)	0 Patients (0%)	2 Patients (6.9%)
Daily (4–7 days/week)	29 Patients (100%)	11 Patients (37.9%)

**Table 3:** Difference in pre‐TIF and post‐TIF scores improvement based on type of TIF (traditional TIF versus cTIF) (*p* < 0.02) (Mann–Whitney Test).

**Improvement in scores after TIF**	**Traditional TIF median (min–max)** **n** = 19	**cTIF median(min-max)** **N** = 10	**p**
Difference in heartburn score between pre‐TIF and post‐TIF	11 (0–21)	21 (0–25)	0.002
Difference in regurgitation score between pre‐TIF and post‐TIF	13 (0–23)	24 (0–29)	0.019
Difference in dysphagia score between pre‐TIF and post‐TIF	0 (−2 to 3)	0 (0–4)	0.839

Two‐thirds of patients (66%) underwent traditional TIF as compared with cTIF (33%). We also found that there was a statistically significant difference (*p* < 0.02) between improvement in GERD‐HRQL, heartburn, and regurgitation scores in our subgroup analysis by comparing outcomes depending on the type of TIF procedure performed (traditional TIF vs. cTIF) (Table 3).

## 4. Discussion

Minor esophageal motility disorders, such as IEM, are a relative contraindication to TIF, so there are limited data regarding their outcomes. IEM can be diagnosed by conventional manometry [5] or high‐resolution manometry (HRM) [6] as seen on the Chicago Classification 3.0 [7]. In a prospective case‐controlled study of 350 patients, about half the patients with IEM had abnormal bolus transit compared to less than 5% in patients with normal esophageal motility [8]. Another US single‐center study examined symptomatology associated with IEM, and dysphagia was the most listed symptom [5]. Our study did not show any statistically significant difference in dysphagia by comparing pre‐TIF and post‐TIF results, which can be a significant practice‐changing finding.

The objective of this study was to assess the short‐term impact of TIF on disease‐specific quality of life and PPI use. Our study showed an improvement in the GERD‐HRQL score without a significant increased risk of dysphagia in patients with IEM that underwent TIF. The median total GERD‐HRQL score (out of 75) was 35 pre‐TIF versus 4 post‐TIF (*p* < 0.001), heartburn score of 20 pre‐TIF versus 3 post‐TIF (*p* < 0.001), regurgitation score of 17 pre‐TIF versus 0 post‐TIF. There was no significant difference in dysphagia between the two groups (median score of zero pre‐TIF and post‐TIF) (*p* = 0.2), which is notable as IEM has often been associated with dysphagia and regurgitation. The secondary outcome measured in our study was PPI use. Although all 29 patients in our study were taking daily PPI before their TIF procedure, only 11 patients (37.9%) were taking daily PPI at 6 months following TIF. Greater than half of the patients (55%) were off PPI. Our study shows that even though TIF is relatively contraindicated in patients with IEM due to the potential side effects of worsening dysphagia and reflux, these symptoms were associated with durable improvements in disease‐specific quality of life with no reported dysphagia after the procedure. Our data positively supports TIF in patients with IEM as a factor that may help change guidelines, as the current recommendations suggest IEM as a relative contraindication to TIF.

The findings of this study are encouraging for patients with IEM, reinforcing the pathophysiological association between GERD and IEM. Chronic reflux has been shown to impair esophageal clearance and disrupt peristaltic integrity, accounting for the frequent coexistence of IEM in patients with longstanding GERD. Although augmentation of the gastroesophageal flap valve and restoration of the antireflux barrier with TIF could theoretically exacerbate dysphagia, the improvement in esophageal peristaltic function observed following reflux control appears to offset this concern.

The data on pharmacological interventions in IEM are limited. Diet, lifestyle modification, and medical GERD management remain the cornerstone of therapy. Besides PPI therapy, prokinetic agents are advised in GERD patients with IEM, with limited data showing benefit [9]. Many studies have shown TIF as an effective treatment choice for patients with GERD symptoms refractory to pharmacological therapy, and it is less invasive compared to laparoscopic Nissen fundoplication (NF) [10]. However, IEM is often excluded from TIF procedure intervention studies [11]. Our study, however, is aimed at examining the short‐term efficacy (6 months) following TIF in patients with IEM and at ascertaining the level of improvement using the validated GERD‐HQRL score.

To our knowledge, only two studies so far have analyzed TIF outcomes in patients with IEM. One study conducted in France has analyzed outcomes of TIF in patients with IEM; the study revealed use of PPI was reduced by 45% in patients who underwent TIF with normal esophageal motility, whereas only 19% of patients with IEM were able to stop PPI use 6 months after TIF [7]. In contrast to the French study, which compared TIF outcomes in patients with IEM versus those with normal motility, our study focused on assessing symptom improvement and quality of life in IEM patients before and after TIF, as it is expected that IEM patients may have more difficult control than normal esophageal motility patients in controlling GERD. Although the French study outlines worse outcomes of TIF in EIM patients, our findings revealed significant quality of life improvement, no notable increase in dysphagia, and a decrease in PPI use after TIF in EIM patients.

Another US‐based prospective cohort study examined the effect of TIF on esophageal motility through high‐resolution esophageal manometry (HREM) or endoluminal function lumen imaging probe (EndoFLIP) before and 6 months after the TIF procedure [12]. They recruited 5 patients with IEM and 11 patients with normal motility. Of the patients with IEM, three out of five (60%) patients showed resolution of IEM, and the remaining two patients had stable IEM. A total of 9 out of 11 patients with normal esophageal motility prior to TIF had normal postprocedure HREM or EndoFLIP at follow‐up. One of the patients developed esophagogastric outlet obstruction on HREM study and another developed diminished‐disordered contractile response; however, both remained asymptomatic clinically. Overall, this study showed median Demeester score decline, though not statistically significant [12]. They concluded the safety of TIF in IEM patients and explored the normalization of esophageal motility in IEM patients after TIF [12]. This study, in conjunction with our study, reveals potential for larger studies assessing not only the safety of TIF in IEM but also potential benefits in reflux symptoms and motility after the treatment of GERD with TIF.

A meta‐analysis found that TIF led to a 54% discontinuation rate of PPIs in GERD patients, with GERD‐HRQL scores dropping from 26 to 6 post‐TIF [13]. Our study specifically targeted patients with EIM rather than all GERD patients and demonstrated similarly favorable outcomes. Given the limited surgical options for IEM patients, as NF is contraindicated, our study highlights the effectiveness of TIF as a viable treatment in this patient cohort.

Our study had limitations, including its retrospective nature and small sample size. This study ended in 2022 after expert relocation from the institute. There was also a lack of objective data, such as a 24‐hour pH study following TIF. The GERD‐HQRL score remains subjective and does not report objective measures such as acid exposure time or improvement in LES competency. The study′s reliance on telephone questionnaires also presents a limitation, as it restricts our ability to evaluate other variables such as diet, weight changes, and other lifestyle changes that may not accurately reflect clinical changes impacting GERD. It is important to note that this retrospective questionnaire may also be subject to recall bias. Future studies should focus on objective measures of changes pre‐TIF and post‐TIF in patients with IEM, such as pH impedance testing and manometry.

Another limitation of this study is the use of the Chicago Classification Version 3.0, which does not incorporate the updates introduced in Version 4.0. Notably, the Chicago 4.0 framework adopts a more stringent definition of IEM, requiring > 70% ineffective swallows and/or ≥ 50% failed swallows for a conclusive diagnosis, and reclassifies fragmented peristalsis within the IEM spectrum [14, 15]. These refinements are intended to reduce overdiagnosis and ensure that patients meeting the diagnostic threshold are more likely to exhibit clinically significant reflux and impaired peristaltic reserve. In addition, Chicago 4.0 outlines a more comprehensive HRM protocol incorporating multiple provocative maneuvers. It is important to note, however, that although this study applied Chicago 3.0 criteria, provocative testing was performed as part of the manometric protocol to better assess contractile reserve and esophageal function.

Despite a relatively small sample size (*N* = 29) and reliance on GERD‐HQRL, our study is comparable to most studies available in the literature, which showed used GERD‐HQRL scores to find a statistically significant difference in outcomes after TIF in normal esophageal motility patients with sample sizes of 28 and 36, respectively [16, 17]. Notably, our inclusion of patients with impaired esophageal motility (IEM) enhances the relevance of our study. Recognizing the limitations inherent to our sample size, we recommend conducting larger studies with extended follow‐up periods to substantiate and validate our findings.

## 5. Conclusion

This study provides valuable insights into the outcomes of TIF in patients with GERD and IEM. Our findings demonstrate that TIF can lead to significant improvements in disease‐specific quality of life, particularly in terms of reducing heartburn and regurgitation symptoms. Additionally, post‐TIF dysphagia did not worsen. Furthermore, a substantial number of patients were able to discontinue or reduce their use of PPIs following the procedure. These results challenge the current perception of performing TIF in IEM, which is considered a relative contraindication, and suggest that it may be a viable treatment option for this specific patient population. Further studies with larger sample sizes need to be performed to validate our results. Overall, this study contributes to expanding the knowledge base on TIF and highlights the potential benefits it offers as a minimally invasive alternative for refractory GERD patients with IEM, potentially avoiding the need for more invasive surgical interventions.

## Conflicts of Interest

The authors declare no conflicts of interest.

## Funding

No funding was received for this manuscript.

## Endnotes

^1^Patients with endoscopically confirmed erosive esophagitis classified as Los Angeles (LA) Grade B, C, and D were included without the requirement of further reflux testing.

^2^Patients with nonerosive reflux disease were confirmed with ambulatory reflux monitoring using either a wireless pH capsule (Bravo) or combined pH‐impedance monitoring.

^3^EsophyX Z+ technology now enables surgeons and gastroenterologists to use a wider selection of standard endoscopes to treat the underlying anatomical cause of GERD.

## Supporting information


**Supporting Information 1** Additional supporting information can be found online in the Supporting Information section. The following supplementary materials will be published alongside the article: Figure S1. Hill Classification for hiatal hernia. Figure S2. EsophyX device: first‐generation and second‐generation devices. This figure illustrates the EsophyX device, depicting both the currently used and newer generation models. Panels A1–A2 show the current device; Panels B1–B2 show the new generation device.

## Data Availability

The data that support the findings of this study are available from the corresponding author upon reasonable request.
