# Supplementary material for: Outcomes in Patients Undergoing Transoral Incisionless Fundoplication With Ineffective Esophageal Motility
Source: Gastroenterol Res Pract. 2025 Dec 22;2025:5054381. doi: 10.1155/grp/5054381 (PMC12752835; doi:10.1155/grp/5054381)
Supplement: Supplementary file 1 — Supporting Information 1 Additional supporting information can be found online in the Supporting Information section. The following supplementary materials will be published alongside the article: Figure S1. Hill Classification for hiatal hernia. Figure S2. EsophyX device: first‐generation and second‐generation devices. This figure illustrates the EsophyX device, depicting both the currently used and newer generation models. Panels A1–A2 show the current device; Panels B1–B2 show the new generation device. [file GRP-2025-5054381-s001.docx]

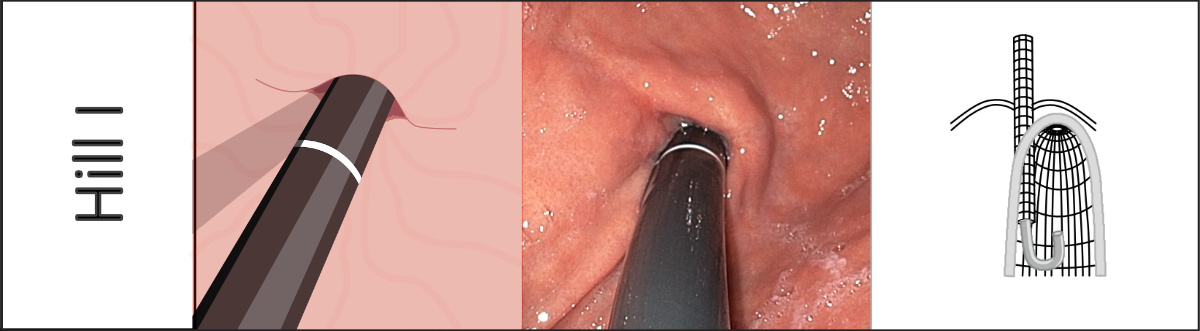

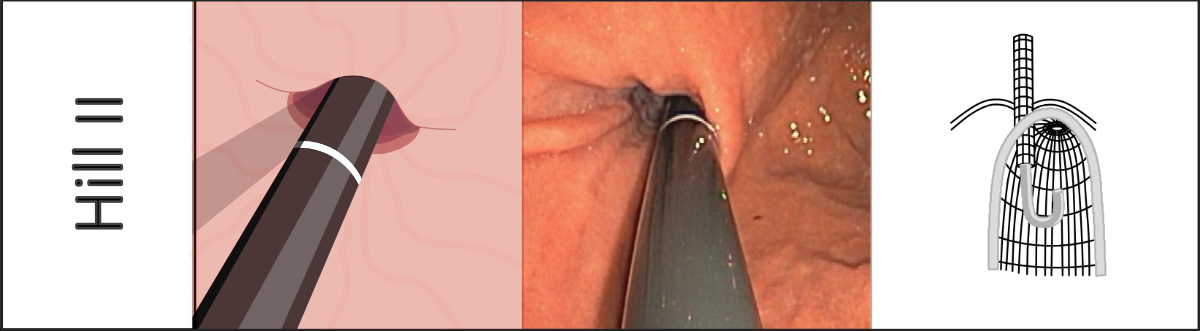

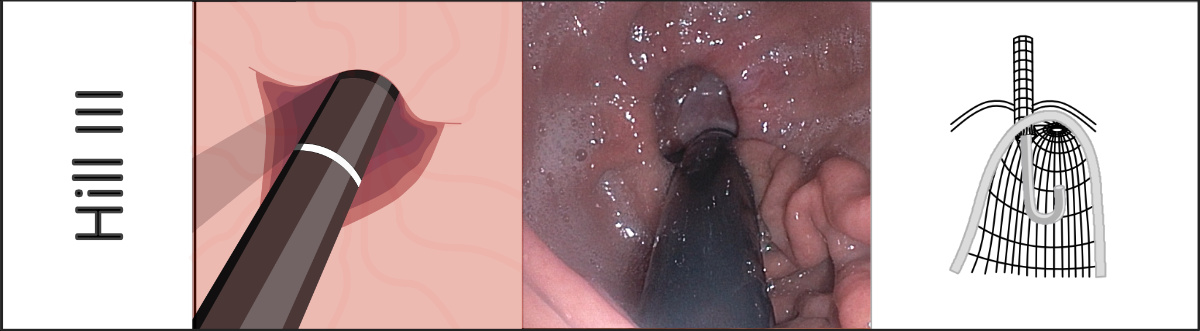

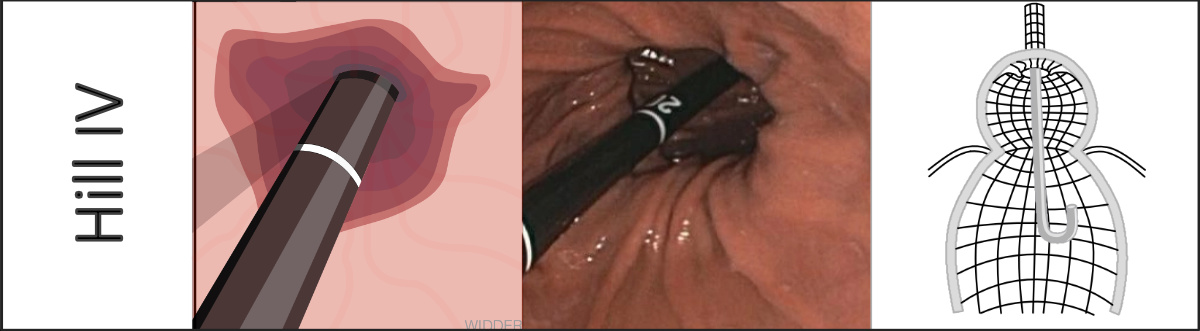

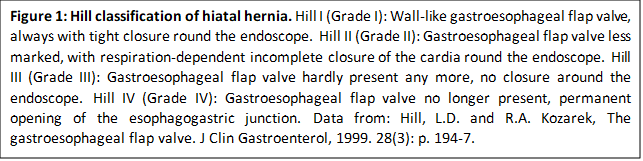


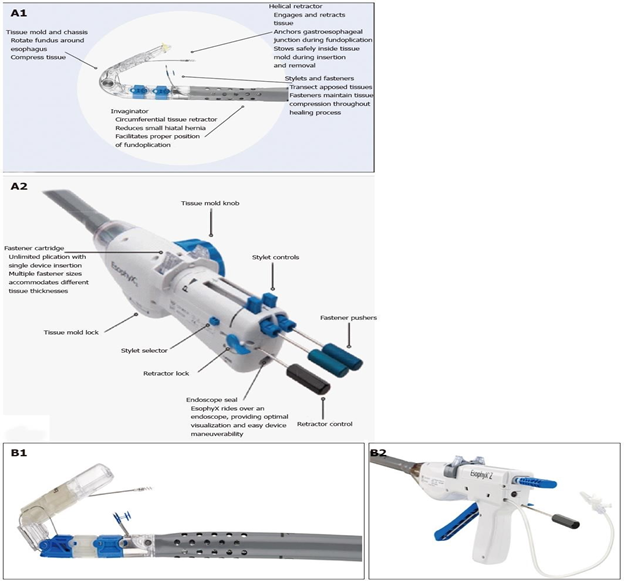


**Figure 2.** EsophyX® device: First- and second-generation devices (courtesy of EndoGastric Solutions, Inc. Redmond, WA, United States). A1-A2: The device currently used (©2014 EndoGastric Solutions, Inc); B1-B2: The new generation device (©2014 EndoGastric Solutions, Inc).
